# Keeping Students with Long-term Conditions Connected with Schools: Facilitators for Sustainable Virtual Connections

**DOI:** 10.5334/cie.133

**Published:** 2024-09-16

**Authors:** Viloshini Baskaran, Laura A. Chubb, Christa B. Fouché

**Affiliations:** 1 Northern Health School, New Zealand; 2 University of Auckland, New Zealand

**Keywords:** long-term conditions, students, social connection, school belonging, inclusion, virtual connection

## Abstract

Students with Long-Term Conditions (LTCs) experience prolonged absences that can impact their school connectedness and belonging. Inclusive education policies in New Zealand advocate for equitable learning opportunities for students with disabilities. Students with LTCs are included under this purview, but responses to their unique learning and connectedness needs are not well articulated. Literature suggests the potential of technology to enable virtual connections to keep these students’ continued presence and engagement in class (i.e., sustainable connections). Studies internationally and in New Zealand, argue that virtual connections with schools can enhance educational opportunities and a sense of belonging for students with LTCs. However, limited research is available on developing and nurturing such ongoing connections with schools. This article reports on a qualitative study investigating New Zealand stakeholder perspectives on the facilitators of virtual connection with schools for students with LTCs. Findings from a thematic analysis of 18 individual interviews with stakeholders highlighted six facilitators for virtually connecting these students with schools, indicating the need for a flexible approach tailored to students’ needs, strong support systems and purposeful, safe and inclusive connection opportunities. The stakeholders indicated these facilitators as essential for the virtual school connections to be sustainable and enhance students with LTCs’ presence, belonging and social connection in schools.

Students with Long-Term Conditions (LTCs) experience prolonged school absences because of their medical conditions. The Ministry of Health New Zealand (2020) regards LTCs as ongoing or recurring conditions such as diabetes, cancers, cardiovascular and respiratory diseases, intellectual disabilities as well as chronic pain that can significantly impact people’s lives. Students with LTCs often receive long-term treatment in hospitals, disrupting their school attendance whilst also risking isolation and exclusion. Healthcare provisions require approaches that can enhance outcomes for people diagnosed with LTCs (Ministry of Health New Zealand, 2020); a population that includes school-aged children and adolescents. Students with special education needs receive support through provisions within the New Zealand educational framework (Mitchell, 2016). As part of this framework, specialist school services are offered to students with individual educational needs (Ministry of Education, 2022). Regional Health Schools (RHS) are one such example, providing ongoing academic support for students with significant health needs in New Zealand.

Being away from school can be complex (Shaw & McCabe, 2008; Shaw et al., 2014) as schooling may be disrupted during hospitalisation due to treatments, attending doctors’ appointments or recuperating at home (Griffith & Doyle, 2009). Students with LTCs can find it difficult to build positive social relationships with peers and teachers when they spend extended time away from school for treatment or recovery. Research indicates that positive relationships and continuity in social relations with others are significant for young people’s health and wellbeing (Allen & Kern, 2017; Child & Lawton, 2019; Goswami, 2012). When students miss out on peer interactions and feel that they do not belong in their classroom, it may negatively impact their academic and social wellbeing including re-entry to school (Goswami, 2012; Weibel et al., 2020).

Literature has documented the importance of the school experience for young people with chronic medical conditions (Kirkpatrick, 2020; Tomberli & Ciucci, 2021). Kirkpatrick’s study investigated perceived school belonging and high school completion for students with chronic health conditions and indicated that they had lower levels of belonging in schools compared to their healthy peers. The author reported that healthy students had higher high school completion rates (Kirkpatrick, 2020). Relationships that foster a supportive learning environment for students with chronic health conditions and opportunities for connection with their classroom appear as fundamental facilitators for their continued education (Capurso & Dennis, 2017; Kirkpatrick, 2020; Tomberli & Ciucci, 2021). Connectedness and school belonging fostered through relationships with peers and teachers are protective factors for young people and, more so, for students with chronic health conditions.

Limited resources and a lack of guidance on effective methods for fostering students’ school belonging can pose challenges for educators to prioritise social relationships for students’ health and wellbeing (Allen & Kern, 2017). For students with LTCs, technology facilitates the connection between the student and the classroom allowing them to have classroom ‘presence’ without needing to be physically present. Discourse on virtual presence in literature includes how immersive technology can enable users to feel real-world experiences of being there in the physical environment (Haans & IJsselsteijn; 2012; Hyun & O’Keefe, 2012; Page et al., 2020). The connection using technology affords these students a virtual presence and inclusion in the classroom that has positive implications for their social connectedness and learning engagement (Powell et al., 2021). A literature review by Baskaran et al. (2022) highlighted the positive implications of connectedness for young people with LTCs within their school environments, facilitated by innovative technologies. The authors stated that research on advanced technologies in school environments is growing concurrent with the innovative use of technology internationally, which includes more recent immersive technologies such as Mobile Robotic Telepresence (MRT). However, this practice only recently gained traction within Australasia for use with young people with LTCs. While studies have shown that the young person’s virtual presence can foster their sense of connectedness with schools (Thabrew et al., 2022; Tomberli & Ciucci, 2021; Yates et al., 2010), very little is known about how such connections can be made sustainable that is, to ensure ongoing presence and engagement in class, in a mainstream school setting (Baskaran et al., 2022).

The reliance on technology to maintain connections has been extensively documented in the literature, gaining central focus during the recent COVID-19 pandemic. While the advantages of sustaining connections between virtual students and their classroom are undeniable, particularly for vulnerable young people, it is also important to address the limitations and challenges associated with using technology for such purposes (Capurso & Roy Boco, 2021; Gilmour et al., 2015; Page et al., 2020; Thabrew et al., 2022).

These studies, along with others, advocate for developing effective strategies to support students’ virtual connections to school and for addressing the limitations encountered in extended online learning periods during the COVID-19 pandemic. This study contributes to the discourse by identifying the key facilitators that sustain virtual connections for students with long-term chronic health conditions. The sustainable connections centralise the virtual student’s ongoing connection to the classroom, which is essential for developing their social network within the school. Being ‘out of sight’ could easily translate to being ‘out of mind’ for most students who experience prolonged school absences due to health. The continued connection with schools facilitated by technology not only supports students with LTCs’ learning but also encourages their connectedness with the school community. Unlike distance learning systems, different considerations are required for this connection to be sustainable for students with LCTs transitioning between school and home or hospital care.

## The Context of LTCs for Young People

An estimated 10% of all young people in New Zealand live with an LTC (Sligo et al., 2019). LTCs can have a significant impact on a young person’s wellbeing. Griffith and Doyle (2009) described such diagnoses as a life-changing experience that alters life trajectories and the realities of being an adolescent.

Inclusive education policies advocate for equitable rights for all young people, including those with disabilities. These policies advocate for young people with special education needs to have access to regular schools that meet their learning needs through a child-centred approach (Hunt, 2020; UNESCO, 1994, 2019). Primarily, what remains a great challenge for both the education and healthcare sectors is to ensure that students with a significant illness are not limited in receiving continued formal education (Gilmour et al., 2015; Page et al., 2020; Slee, 2019; Sligo et al., 2019).

For students with LTCs, schooling is disrupted while they are hospitalised, attending doctors’ appointments, or recuperating at home (Griffith & Doyle, 2009). Pate et al. (2017) found that having a connection with school acted as a protective factor for young people. Diagnosis with LTCs can have a profound impact on the young person’s self-perception. Sligo et al. (2019) reported that participants in their study described having a fear of being unwell, resentment for missing out on experiences that other young people their age had, and trauma from the LTC experience. Spencer et al.’s (2018) study, conducted at a university in Australia, observed that students with chronic health conditions sought to position themselves as healthy, but were challenged to keep up the healthy narrative in university settings. Not having the energy to manage complicated administrative processes resulted in some young people preferring not to seek help and instead concealing their health needs. Findings from the study highlighted that understanding and awareness of the chronic condition experienced by young people is needed.

In addition to ensuring equitable learning opportunities, schools must be capable of receiving and supporting students with LTCs. In New Zealand, RHS enrol students when they are unable to attend school due to illness. To date, there is a paucity of research on the impact of RHS on supporting the wellbeing of their students. Hopkins (2015, p. 17) investigated the nature of education support provided for students with health conditions across Australia and New Zealand and reported on the ‘fragmented’ but also ‘uncoordinated’ learning structures based on diversified models. Recommendations from the report stated that educational support models for students with LTCs should provide emotional and social support along with opportunities for peer connectedness (Hopkins, 2015).

## Virtual Connection with Schools for Students with LTCs

Research has shown that students with LTCs are disrupted academically, socially (often facing challenges in forming strong relationships with peers) and emotionally when absent from school (Gilmour et al., 2015; Pate et al., 2017; Shiu, 2001). Children’s subjective wellbeing is greatly impacted through positive friendships (Goswami, 2012). Pate et al. (2017) reported that adolescents who felt socially connected with their schools had higher academic performance and completed more years of schooling. It is not about having larger social networks, but the quality of the relationship that is important (Child & Lawton, 2019).

As a starting point to shape a New Zealand response that fosters connections disrupted by illness, a literature synthesis was conducted to collate insights from existing studies and policies relating to social connectedness through technology for young people with LTCs (Baskaran et al., 2022). The findings evidenced sparse research from the New Zealand context and the need to involve policymakers, teachers, doctors, and other key stakeholders working with young people as well as young people themselves in exploring what will help improve current practices to support students with LTCs’ sense of belonging in schools.

New Zealand researchers pioneered the Patience Project to foster social inclusion for home-or-hospital-bound students with a significant health condition using telepresence technology. The initiative reported positive results where students described an improved sense of belonging and wellbeing (Chubb et al., 2021; Thabrew et al., 2022). The Patience Project trialled an intervention using a virtual reality headset that allowed the student to navigate and view 360-degree live-streamed images of the classroom (Chubb et al., 2021). The students in the study also completed the Short Warwick-Edinburgh Mental Well-Being Scale and the Social Inclusion Scale, and the quantitative data similarly reported improved social inclusion, connectedness and wellbeing (Thabrew et al., 2022). Participant responses indicated improvements to students’ wellbeing using telepresence technology for connection, enabling participation in class activities, and interaction with their peers or teachers (Chubb et al., 2021; Thabrew et al., 2022).

Advancement in technologies have led to innovative possibilities for establishing connections with classrooms. Newhart and colleagues used focus groups in classrooms and observations to investigate outcomes for students with significant illnesses when connecting with classrooms using telepresence robots (Newhart & Olson, 2019; Newhart et al., 2016). Similar studies testing various technologies demonstrated successful outcomes for learners unable to be physically present in class (Soares et al., 2017; Weibel et al., 2020). As such, virtual inclusion initiatives have progressed conversations around the significance of keeping young people with LTCs present in classrooms (Baskaran et al., 2022).

Studies investigating social and school connections for young people with LTCs indicated the significance of maintaining social connectedness to improve their wellbeing and sense of belonging (Hopkins et al., 2014; Maor & Mitchem, 2020; Yates et al., 2010). Engagement with schools to foster or maintain social connectedness can mitigate the issue of isolation for these young people (Hopkins et al., 2014). The opportunity for school connection using technology can support their continued education, engagement, and social interactions in the classroom.

In New Zealand, the focus on virtual inclusion initiatives for social connection is a burgeoning area of study where students with LTCs are concerned, including students’ perspectives to develop insights into virtual initiatives that can better support connection has only recently been documented (Chubb et al., 2021; Thabrew et al., 2022). However, investigations using collective stakeholder perspectives are still rare. Findings from the research reported in this article include multiple stakeholder perspectives from New Zealand, including those of young people who previously had an LTC, about potential facilitators for advancing virtual connections with schools.

## The Present Study

This article reports on a qualitative study that explored New Zealand stakeholder perspectives on facilitators for virtual connections with schools for students with LTCs. This study refers to *virtual connection with schools* as connecting the students with LTCs in the classroom using technology to provide opportunities for them to participate in learning with their peers and develop social connectedness and school belonging. Studies have documented the positive implications of using various technologies for connecting students with LTCs in their classrooms including more advanced immersive technologies such as telepresence robots (Chubb et al., 2021; Gilmour et al., 2015; Thabrew et al., 2022). This study discusses the implications and the ways to enhance effective connections for the student and to support their online presence and inclusion in schools. The term *virtual inclusion* found in other research (see Newhart & Olson, 2019; Newhart et al., 2016) suggests that virtual connections allow students to be present and feel included in their classroom. Students with LTCs’ virtual presence can support their participation in lessons and engagement with teachers and peers as if they were physically present in the classroom. The findings from this study can offer guidance to school leaders and support workers on how they can facilitate the students with LTCs’ school belonging and connectedness through virtual school connections.

## Method

### Participants

The article reports on an exploratory qualitative study investigating the facilitators of virtual connections with schools for students with LTCs. The study intended to identify facilitators that support the virtual connections for students with LTCs in schools. Ethics approval was granted (University of Auckland Human Participants Ethics Committee/ Reference UAHPEC2772). Eighteen research participants were recruited through a nonprobability purposive sampling strategy. Key stakeholders were selected based on their experiences with LTCs or supporting students with LTCs. Stakeholders included family members, teachers, support workers, medical professionals, and young people with LTC experience. Table 1 presents the participant list in the order of their interview. These participants were four young people, aged 16 and above, with experience of LTCs; four RHS teachers and a senior leader; one school counsellor; two healthcare professionals; one academic from a tertiary institution specialising in inclusive education; four teachers involved in a school connection initiative using virtual technology (including the Patience Project operations manager); and one parent. A sample representing diverse stakeholder perspectives was significant to this study. Participation was voluntary, with stakeholders contacted through a snowball approach and recruited via third-person support. A pseudonym is used for each participant.

**Table 1:** Description of the Stakeholders Interviewed.


NO.	PSEUDONYM	ROLE

1	Aqua	Community teacher, Regional Health School

2	Saphire	Patience Project operations manager

3	Cadet	Clinical psychologist (Consult Liaison Service) with experience supporting students with LTCs

4	Sage	Community teacher, Regional Health School

5	Frostblue	Haemodialysis educator and professional leader with experience supporting students with LTCs

6	Jade	Senior leader, Regional Health School

7	Freya	Community teacher, Regional Health School

8	Neva	Community teacher, Regional Health School

9	Aero	Young Person with LTC experience and CanTeen NZ Ambassador (school leaver)

10	June	University Senior Academic, Inclusive Education

11	Killarney	Mainstream school teacher; participated in the Patience Project

12	Sam	Mainstream school teacher; participated in the Patience Project

13	Azure	Parent, whose child attends a Regional Health School; participated in the Patience Project

14	Damon	Young person with LTC experience and CanTeen NZ Ambassador (school leaver)

15	Lilac	Young person with LTC experience and CanTeen NZ Ambassador (school leaver)

16	Auburn	College counsellor; participated in the Patience Project

17	Mauve	Young person with LTC experience; participated in the Patience Project (high-school student)

18	Jasmine	Mainstream school teacher and dean; participated in the Patience Project


### Design

The stakeholders participated in individual semi-structured interviews that lasted one hour on average. All interviews, except one, were conducted online due to the pandemic restrictions. Based on a comprehensive literature review, key areas for the interview guide were identified and pilot-tested (Baskaran et al., 2022). Open-ended question prompts were designed for these areas, including: the significance of social connectedness for young people with LTCs; support systems within their school or community to keep young people with LTCs connected; interactive technologies that support student and school connection; and enablers and limitations for using these interactive technologies for connection.

Studies indicated limited understanding of this subject in New Zealand (Baskaran et al., 2022; Chubb et al., 2021; Thabrew et al., 2022). Therefore, the semi-structured interviews provided the opportunity to engage with the stakeholders in a rich discourse to gather insights on the facilitators that support sustainable virtual connections for students with LTCs and their schools.

### Analysis

Audio recordings were transcribed verbatim, and the first analysis cycle was performed on all 18 interview transcripts by the first author. Data were analysed using Braun and Clarke’s (2006) six-step thematic analysis. After becoming familiar with the data by reading and re-reading the transcripts, the first author coded data at a semantic level in this first analysis stage. NVivo, a computer-assisted qualitative data analysis software, was used to analyse and code the transcripts using a top-down approach. In the second analysis stage, these codes were interpreted to find patterns across the codes. Recurring codes were placed under candidate categories. From the emerging patterns, the authors identified key facilitators for maintaining student and school virtual connections, with particular considerations for students’ experience with long-term health conditions. The codes were then re-examined and compared against the dataset to ensure none were left out. The key categories were finalised and reported after the authors discussed and resolved any discrepancies.

## Findings

Participants agreed that creating virtual connections with schools is important for advancing the social connectedness of students with LTCs. However, for this initiative to be effective, it is important to understand the physical and psychological demands students with LTCs experience at this time in their lives. The findings from this study indicated that the connection between students and the school can be effective if made sustainable through an initiative that supports their ongoing presence and connectedness with schools. From the interview data, six facilitators were identified to foster sustainable connections with schools for students with LTCs. These conditions are summarised in Table 2 and discussed in more detail in this section.

**Table 2:** Facilitators for Sustainable Virtual Connections Identified from the Interview Data.


FACILITATORS	DESCRIPTION

Individual student needs	⚬ flexibility to include virtual connections in learning environments based on students’ needs ⚬ empowering students in the decision making

Culturally responsive support	⚬ understanding students’ and their families’ values and beliefs ⚬ culturally competent practices for virtual connections

Purposeful virtual connections with schools	⚬ making virtual students feel present and included ⚬ mitigating exclusion through purposeful connections

Technology for virtual school connections	⚬ positive inclusive experience for students that supports their social connectedness ⚬ managing expectations and addressing concerns

Safe online environments	⚬ privacy in online environments ⚬ students feel safe in the virtual connection

Stakeholder training and support	⚬ health literacy for support providers ⚬ accessing and receiving support ⚬ developing teacher preparedness


Findings indicated that communication between the young person and family members with RHS teachers and mainstream school teachers occurred online, supplemented with face-to-face home or hospital visits. Participants reported that students with LTCs spend significant time engaging in online learning with support from their RHS teachers. Other studies have reported on hospitalised young people’s prevalent use of technology to disrupt their isolation and remain connected with family and friends (Maor & Mitchem, 2020; Thabrew et al., 2022). Considering the health of these students, the stakeholders understood that using technology was a viable option to keep them virtually connected with their schools. They stated that to keep the students with LTCs connected with schools effectively, the connection needs to be ongoing and not a one-off initiative.

Identifying the facilitators to maintain these students’ virtual connection to schools is a step towards recognising the importance of school presence and participation for their social connectedness and wellbeing. Incorporating these facilitators in the design and implementation of virtual connection initiatives can enhance the sustainability of keeping students with LTCs’ connected to their schools.

### Individual Student Needs

The first facilitator, as outlined in Table 2, pointed to the importance of prioritising students’ needs. Several stakeholders saw this as crucial to ensure the feasibility of virtual connection to improve social connection. Students with LTCs have unique needs based on their motivation, personality, and health on any given day. Listening and responding to their needs is important because of “their different experiences…and spaces that people are in” (Sage, RHS Teacher). Killarney’s (school teacher) experience with using virtual technologies in school exemplified how the student preferred being seen in class. The teacher and classmates wanted to be able to see him through the camera, but “he just wanted to see us.” “There would have to be some flexibility there to accommodate whatever concern they have,” said Neva (RHS teacher). The type of technology or the approach “has to be modified to the person” so that they have an option to identify what their need really is (Cadet, clinical psychologist).

Amplifying students’ voices is key to addressing their individual needs. Damon (young person) stated that asking students, including young children, “what they actually want” is crucial to prioritising their needs. Sapphire (Patience Project operations manager) described giving the students “a sense of control back” in terms of “how often they stream in and become part of that classroom is entirely up to them.” For students experiencing LTC, that sense of control and “having some say in how it works for them” (Sam, school teacher) welcomes normality back into their lives.

RHSs have a practice in place through their Individual Learning Plans (ILP). ILPs are designed to meet the learning needs of their students with medical conditions. However, challenges in the education system and school infrastructure form roadblocks that impede the flexibility to prioritise students’ needs, as stated by Freya (RHS teacher), “it is not only listening to what they want, but it is trying to act on that, which can be hard. Some of those barriers are pretty hard to move in the education system”.

To address some of the challenges in prioritising individuality, June (academic) suggested that students be empowered to be part of the decision-making process so that their needs and those of their families are more clearly articulated. June emphasised that;

we should then be ensuring that we listen as professionals … and then the next step is we should be ensuring we respond … to what the children have said and question ourselves about what needs to change in order to support them well.

### Culturally Responsive Support

The second facilitator for sustainable virtual connections with schools indicated the need for stakeholder support to be culturally responsive, which extends to how schools manage the virtual connections made for students with LTCs. To invite long-term connections that do not lose their value once the novelty of technology wears off, schools should understand how virtual connections will be treated and used based on the student and their family’s values and beliefs.

Freya (RHS teacher) described how bridging this culturally responsive support, whilst necessary, can be quite hard to achieve for families of different ethnicities and cultural values. As a healthcare professional, Cadet (clinical psychologist) noted similar observations through his experience treating patients. Not approaching the available support services could be driven by “fear” of not knowing how to access them. To further compound this situation, the socioeconomic background of families can impact how they perceive and receive support services. Support services and providers thus need to apply culturally competent practices, but not everyone knows how best to deliver these. Sage (RHS teacher) reflected on an experience supporting a student:

I have the absolute best of intentions and wanting to help her, but I do feel really inadequate and not knowing enough about her culture and how to … access the family in a way that is going to be meaningful.

Each stakeholder wants what is best for the students with LTCs and will try, to their best ability, to meet their interests and needs. Sage expressed that responding to the students and their family’s needs in a culturally responsive way is “definitely more effective.”

Participants articulated that schools and teachers try to drive student- and family-led approaches when providing support for school connection “because we have to base it on their values and what their goals are” (Freya, RHS teacher). June (academic) added that a foundation for culturally responsive practices can be built through good relationships where the teacher “has good support and understanding around what that means for their role” to ensure “those relationships are maintained and kept really strong.”

### Purposeful Virtual Connections with Schools

The third facilitator identified that students with LTCs need to feel present and included through a purposeful connection with their school. This connection is significant for these students who might feel singled out when all their other peers are physically present in the classroom while they are not. The initiative for sustainable virtual connection will be a rewarding experience when it is “receptive on the other end” of the classroom (Aqua, RHS teacher). The realities of the young person being unwell at the time of the connection should be seriously considered. This means prioritising purposeful connections that enhance students’ sense of presence and belonging.

Jade (RHS senior leader) offered an example of where students can lack the motivation to participate when they feel excluded from the lesson:

She said that when she joined the class, it was cool the first couple of times, but then after that, she said … all that would be happening is she would be looking at them with their heads down, working on their books … it wasn’t as if she was joining in to do the work. They need to feel like they were part of the lesson.

Aqua (RHS teacher) emphasised that making the student feel included is important, and this means considering, “the purpose of the technology; what we are trying to achieve; and how best to facilitate that.” Jasmine (school teacher) suggested active interaction to purposefully connect to the student:

I always started off by greeting her, as I would if she walked through the doors … and then I would go about my normal everyday lesson. The girls would go up to the camera, they would talk to her … as she became more familiar with it, she would be a lot more engaged. Just being familiar, feeling at ease… of doing the same thing 4 hours a week.

Being purposefully connected mitigates the sense of exclusion often experienced by these students. This sense of exclusion, however, can be heightened when teachers lack the skills and time to actively engage with the student connected by camera. Aero (young person) shared that students can experience the disconnect in terms of “I am not there with you one on one, even though we are one on one right in their screen right now.” Lilac (young person) expressed the challenge for teachers “to pay attention to someone on a screen when everyone else is there in person,” adding that the student “could almost get pushed to the back”.

To encourage their students’ virtual presence, teachers and in-class peers should collaboratively make the connection purposeful. Teachers can rely on their skills and training to keep students engaged, by knowing how “to include them and how to draw them out” (Auburn, school counsellor). It is important that “what teachers do in their classroom does include everybody” (June, academic). June pointed out that teachers consider their “teaching practice, to think about how we can plan and teach with everyone in mind from the outset.” Teachers should consider how the technology operates in the classroom and how the student can feel included in lesson activities through this virtual connection.

### Technology for Virtual School Connections

The next facilitator signals that technologies can support virtual connections with schools. However, how this medium of connection effectively supports students’ connectedness and inclusion in the classroom should be considered. The stakeholders cited Google Classroom, Microsoft Teams and, more recently, Zoom teleconferencing as common platforms used in schools to enable virtual interactions for students with LTCs. Connecting to school through virtual reality technology has also become a possibility.

The young people in this study viewed the connection with schools using technology as advantageous. Aero expressed that he would have “taken huge advantage” of such technology, and Lilac shared that it would have been “cool” to “see all my friends…still being able to be in that environment.” Damon concurred that virtual reality technology “would be amazing to use … feeling that you are actually in a classroom.” Similarly, Mauve (young person), who experienced a virtual connection using telepresence technology, shared that transitioning back to school was easier as “I feel like I have been here this whole time.”

Some participants, however, thought using technology was *assistive* but should not be *replacive*. Freya (RHS teacher) commented on the “social aspect of learning and wellbeing because that is so easily overlooked,” adding that for students with LTCs, “people assume there is a lot of socialising because of social media … it is a different type of socialising; it is not face to face.” Freya shared that face-to-face interactions cannot be replaced, but “you could improve on digital interactions to make things more real” for the virtual connection with schools.

How students connect and communicate through virtual platforms can vary, especially for different age groups. Neva (RHS teacher) added that when the teacher facilitates communication and connection, this opens the opportunity for “inclusion for everybody rather than just the popular kid or the confident kid.” A real challenge, however, can be for teachers to provide a positive inclusive experience that enhances the virtual student’s sense of connectedness with the classroom. Tasks carried out in the classroom should be achievable for the students connecting from hospital or their home. Managing these expectations is a step toward ensuring successful continuing connections for the young person with LTC. Realistic expectations from all parties involved and collaboration are essential to ensure that the teacher is comfortable supporting the connection and is aware of the plans made. Teachers should feel confident using the technology and be guided in ways to make students on screens feel included in the class. Neva shared one time when a lower primary student did a show-and-tell lesson streamed into class as part of question time. It “went really well because the teacher was comfortable with including the student in that part of the lesson.”

Concerns about using technologies and the challenges of supporting social interactions through technology can be mitigated through transparent communication. Teachers who are already overburdened by teaching tasks need to feel they will be supported if they are going to create virtual connections for students who are unable to attend school. Ideally, key stakeholders working collaboratively to support each other would ensure a higher chance of the virtual connection with schools being impactful. Sage (RHS teacher) shared this experience, “I have had good success with kids … when the medical person, the school and their family are all working together for the same goal, all on the same page.”

Participants commented that bringing stakeholders together to work collaboratively can be challenging, but the benefits outweigh the challenges for the young person who needs the support.

### Safe Online Environments

The next facilitator points to the importance of creating a safe online environment to address valid privacy concerns. The privacy issue is a concern not only for students with LTC but for teachers who might feel they are under surveillance when the camera is placed in the classroom. One key concern surrounding privacy is the use of cameras in the classroom:

There is a vulnerability to having your class being accessed from outside and because teachers are often on the receiving end of … harsh criticism and judgment … students [are] perhaps screenshot-ing or recording and even using it for nefarious purposes (Sage, RHS teacher).

From a school management perspective, Jade (RHS senior leader) commented, “I can understand why some teachers would feel very reluctant to have a camera on them. It would be quite intimidating.”

Whilst some RHS teachers were concerned about privacy issues for students with medical conditions when the camera is turned on, others felt that this concern can be addressed with careful planning. Teachers shared that students in class and their parents usually encourage the connections that can benefit the student who is absent. Killarney (school teacher) shared, “we came from the perspective that we are privileged to try out this new technology, so everyone was quite keen.”

Similarly, Jasmine, who trialled the virtual technology for their student, expressed that the other students in the class felt excited “to see the student every lesson. They felt like they were involved.”

Students, too, need to feel safe in this online environment, especially if schools provide it. Freya (RHS teacher) emphasised, “we want our students to understand that they can come here and we are going to make sure this is safe for them.”

The teachers’ concerns included protecting the privacy of the young person and their family. Importantly, the participants shared that creating a safe online space is necessary, and precautionary steps, “putting those safety networks [in place]” (Frostblue, haemodialysis educator), should mitigate the risks.

Damon, a young person with LTC experience, shared that one way to address the privacy issue is through open communication with the individual, giving them the opportunity to voice their needs, “the person might be 10 years old, but they still have feelings, they still know what they want.” Cadet (clinical psychologist) concurred that “privacy is always a very valid concern,” and participants noted the importance of transparent communication and collaborative knowledge sharing to address this real challenge.

### Stakeholder Training and Support

The final facilitator to sustaining virtual connections with schools for students with LTCs is to ensure the stakeholders are trained and supported. Findings revealed three areas to enhance stakeholder training and support. First, stakeholders pointed to the importance of health literacy for support providers to support students with LTCs and their families effectively.

Students with LTC require attention for their health care. Sustainable connections become viable when support providers are equipped with literacy concerning students’ healthcare needs, as pointed out by Cadet (clinical psychologist), “if you carefully stage-manage what it is like to have chronic illness and how beneficial this technology will be … that leads to more outcomes … health literacy is a big component.”

Similarly, Neva (RHS teacher) noted the importance of teachers’ understanding of their students’ unique health needs. If they “unfortunately do not see that as a real health problem … or they are not comfortable discussing them … it just gets swept under the carpet.”

Second, the sustainability of the connections becomes viable when students and their families feel they can seek and receive the support offered. To achieve this, stakeholder training and support are crucial. When services can be delivered effectively, students with LTCs and their families feel supported. One participant praised their child’s school, stating that “the school and Health school are amazing. Between everybody that is involved, I feel very supported academically and socially” (Azure, parent to a child with LTC).

However, the experience of being supported is not the same for everyone. Damon (young person) shared the sense of detachment:

Going back to school after treatment … was as if nothing had happened in the first place. Looking back, I do wish that both my brothers and I had more support during my treatment and posttreatment as it was quite difficult to get back into the swing of things.

Third, stakeholders shared that supporting students with LTCs in their social interactions in schools should be given more focus. Aqua (RHS teacher) expressed that this support is “pivotal to wellness and getting back to school,” adding “opportunities are limited, and it is a challenge to provide them.”

When roadblocks to educational systems and school practices are present, connections with schools for students with LTCs are put under the radar. Professional development for teachers mostly focuses on using technology for learning. Freya (RHS teacher) shared that professional development focuses on “the use of technology, not so much related to the social aspects of learning and development.”

One way to address the roadblocks is to ensure teacher preparedness through efficient support and training. Teachers strive to provide the specialised care these students need, but as Sage (RHS teacher) explained, the barrier of teacher fatigue in dealing with ongoing issues are tricky to resolve. Teachers can feel supported in their efforts to establish connections for their students with LTCs when they are part of knowledge-sharing practices on healthcare needs and understand the purpose of connection.

Findings from the study pointed to the need for teachers to be trained and supported to keep their students with LTCs included and connected to the classroom virtually:

Systems do not always support the teacher to be able to participate fully in the kinds of conversations that need to happen around supporting that child to belong, to have friends, to learn well and to keep that connection with school. (June, academic)

June added that providing professionals with time is crucial for them “to come together as needed” and “share their knowledge and to work … interprofessionally for the benefit of the child.”

## Discussion

In New Zealand, students are supported by their RHS teachers to transition back into school after being absent due to an LTC. However, maintaining an ongoing connection between the young person and their schools is not currently a priority in mainstream schools. Fostering sustainable virtual connections (i.e., ongoing and consistently available) for students with LTCs is timely. Further, the global shift to distance learning during the pandemic showed the potential and limitations of technology for education. This period demonstrated the need for adaptable technological solutions for young people with special education needs to maintain inclusion and equitable access to education (Capurso & Roy Boco, 2021; Usher et al., 2020). Several studies provided an understanding of how technologies can be used to create purposeful connections for hospitalised or home-bound students and ways to address the limitations of ICT for such connections (Chubb et al., 2021; Maor & Mitchem, 2020; Newhart & Olson, 2019; Page et al., 2020; Soares et al., 2017; Thabrew et al., 2022; Weibel et al., 2020). The possibilities for sustainable virtual inclusion for these students are encouraging and point toward advantageous outcomes for their academic, social, and emotional wellbeing.

Understanding how schools can support these sustainable virtual connections for the young person with an LTC to continue having presence and engagement in class is an important step in enhancing students with LTCs’ sense of belonging and social connectedness with schools. The six facilitators discussed by stakeholders, including students with LTC experience, are a solid starting point to initiate the practice of virtually including students with LTCs in New Zealand classrooms, with a need to provide sustainable and continuous connection. This means opportunities to connect must be present in schools to support the presence and participation of students with prolonged school absences due to significant illness. Connecting should be driven by the young person’s capacity given their health. Choosing when, where and how to connect is essential for the young person to establish a sense of connection with schools (Thabrew et al., 2022).

Due to illness and transitioning back, being away from school can be a complex process for students with healthcare needs (Shaw & McCabe, 2008). Supporting the presence and belonging in school of students with LTCs is critical. However, for this support to be effective and purposeful, school stakeholders must understand the physical and psychological demands placed on students at this time in their lives. Differentiating students based on their ability to be present and participate in class activities can stem differentiation and deficit for students with disabilities (MacArthur & Rutherford, 2016).

The stakeholders in this study emphasised the need to account for, and respond individually, to the needs of students and their families, for the virtual connection with schools to be effective. Responding to the individual needs of students with disabilities is integral to inclusive education policies. Individual Learning Plans are developed for students in RHSs in New Zealand (Hopkins, 2015). The ILP includes transition plans that support the student’s transition back to school. Integrating individual needs for virtual connections with schools in these ILPs would be advantageous to sustain the connection with remote students. All stakeholders called for students with LTCs to be active in the decision-making for their virtual connections with schools. As students and their families have diverse values and beliefs about school connections using technologies, support responses must be culturally competent in meeting their needs.

Research shows that a sense of normalcy is what these students need to escape isolation (Chubb et al., 2021; Sligo et al., 2019). A continued connection with their school environment can protect their social integration (Shaw et al., 2014) and emotional wellbeing (Pate et al., 2017; Shiu, 2001; Thabrew et al., 2022). The findings in this study point to virtual connections with schools being ongoing and long-term as an important facilitator for students with LTCs to feel socially connected and included in the classroom. The stakeholders described as a key consideration how lessons and tasks should be designed so that students connecting remotely feel included in a blended learning environment and how teachers can be supported in their classroom practice (Newhart & Olson, 2019).

Students who cannot attend school, slowly transition to being ‘out of sight, out of mind’, supporting findings that realistic expectations for the use of technology for connection based on the student’s individual needs must be set to enable their continued presence and engagement in class virtually (Weibel et al., 2020). Further, stakeholders in this study agreed that the support systems would be strengthened by fostering virtual connections with schools where the environment feels safe for students and their privacy concerns are addressed. Newhart et al. (2016) pointed to a lack of guidelines to help teachers and schools facilitate the virtual inclusion of students with significant illness in the classroom. As highlighted in other studies, teacher preparedness to use and navigate the technology during the connection is central to ensuring that such support meet the needs of these students (Newhart et al., 2016; Weibel et al., 2020).

A young person’s sense of self, self-efficacy and social relationships are impacted by illness, but positive interventions can aid their adjustment (Beeman & Henderson, 2012; Shiu, 2001). Findings from our study evidenced the potential of a positive intervention that can provide continuity of learning and social interactions for these students through their virtual connection with schools. To make this possible, support providers must fully understand the impact of LTCs on students and the adverse implications they experience from being isolated from their school environment. Mukherjee et al. (2000) stated that teacher responses can range from empathy and encouragement to a lack of flexibility and sympathy. Building on interpersonal collaboration can strengthen relationships between stakeholders, promoting the transfer of knowledge to build stakeholders’ health literacy and fortify their capacity to support students with LTCs within both health and educational contexts (Hopkins et al., 2014; Mukherjee et al., 2000). The findings indicate that health literacy is fundamental to preparing stakeholders to support these students through their schooling experiences. Health literacy can be made possible through interprofessional collaboration.

Teachers in our study were supportive of virtual connections with schools that support these students’ sense of connectedness and belonging to the classroom, but teacher preparedness is central to its success (Newhart et al., 2016). Not only do teachers need to be comfortable and confident in using the technology to support the students’ connection with schools, but they also need to be flexible in their teaching approach to meet the learning needs of their in-class students and virtual students. Teachers need to consider how they can adapt learning tasks to encourage their virtual students’ participation. Supporting teachers is important, as is managing the expectations of stakeholders involved in the initiative.

Although New Zealand is at the forefront of inclusive education practices, more can be done in schools to meet the needs of priority learners with LTCs (Barback, 2018; Sligo et al., 2019). Findings from this study reinforce the importance of stakeholder perspectives, including the voices of young people with LTCs, to inform the design of initiatives that can support school connections for students with LTCs while away from school and on re-entry after hospitalisation. The visibility of young people, prioritising their participation and inviting them to lead decision making for themselves, is fundamental to exercising their agency and rights (MacArthur et al., 2018).

## Conclusion

Sustainable virtual connections (i.e., ongoing and consistently available) offer students with LTCs opportunities to connect to school on their terms. Educators and policymakers should consider sustainable connection as a key concept when advocating for initiatives that can connect students with an LTC to their schools, given the flexibility and student-driven nature of virtual mediums for connection. The findings demonstrate that sustainable connections with schools show promise for students with LTCs to maintain a continued sense of school belonging.

The shift in learning pedagogies to support students’ learning during the pandemic has paved innovative teaching methods that advocate not only inclusiveness but also adaptiveness and resilience. However, the consequential negative impact and inequalities exacerbated through this shift are important considerations. Issues such as digital equity, privacy concerns, and the risk of reduced face-to-face interactions must be carefully managed (Gilmour et al, 2015; Thabrew et al., 2022).

Burgeoning literature offers guidance on developing effective practices to support students’ schooling and online learning, especially on ways to cope with the psychological challenges of losing important face-to-face interactions in a school setting (Capurso & Roy Boco, 2021; Smith & Lim, 2020; Usher et al., 2020). Adding to this discussion, this study advocates continuous connections offered by technology for students with chronic health conditions who may experience prolonged school absenteeism.

Application of the facilitators for the sustainable virtual connections highlighted in this study may facilitate these students’ reintegration into schools more rapidly. More importantly, ongoing connections with the school environment can establish a sense of normalcy for these students. Feeling included and that they belong through virtual initiatives may provide meaningful opportunities for students with LTCs to engage in learning and social interactions that can positively impact their overall wellbeing. Virtually connecting students with LTCs in schools enables visibility of healthcare needs that would otherwise have been masked through their extended time away from school.

## Limits of the study

This study involved a diverse range of stakeholders, thus significantly enriching the breadth of perspectives on virtual connections with schools for students with LTCs. However, this study’s main limitation was the low recruitment of participants during the COVID-19 pandemic. The limited participation of young people with LTCs resulted in a restricted scope of findings regarding their perspectives on virtually connecting with schools. Nonetheless, it is evident that the stakeholders involved in this study recognised the benefits of initiatives that promote social connectedness between these young individuals and their schools. Conducting further studies on the perspectives of young people with lived experiences of LTCs, across various age groups, on connecting to schools virtually and how they would want this connection to be sustained, will add useful data to enhance the sustainability of this practice in schools.

## Data Accessibility Statement

The data that support the findings of this study are available on request from the corresponding author, [VB].

## Author Information

Dr V. Baskaran is an early career researcher and educator. Her PhD research focused on virtual inclusion initiatives to connect young people with long-term health conditions with schools using interactive technology. Her research expertise is in supporting students’ school belonging and transitions. She is interested in Manaakitanga (care and compassion) in schools’ inclusive practices, children’s inclusion in research, and the development of educational technology for learning and school connections.

Dr L. A. Chubb is a researcher and Senior Lecturer at the University of Auckland, New Zealand, specialising in global health and community development. Her research expertise centres on strategies to enhance young people’s well-being through co-creating accountable spaces for sensitive discussions and futures-thinking. She has extensive engagement with communities internationally in Kenya, Ghana, and New Zealand to advance an interdisciplinary focus on young people’s wellbeing.

C. B. Fouché is a Professor of Social Work and Associate Dean (Postgraduate Research) in the Faculty of Education and Social Work, University of Auckland, New Zealand. Christa is an applied researcher with expertise in health and social care workforce development and in designing and implementing interdisciplinary and culturally diverse community-based projects.
